# THE IMPACT OF SOCIAL INEQUALITIES ON DIGITAL ENGAGEMENT OF OLDER ADULTS DURING THE COVID-19 PANDEMIC

**DOI:** 10.1093/geroni/igac059.110

**Published:** 2022-12-20

**Authors:** Andraž Petrovčič, Darja Grošelj, Štěpán Žádník, Katja Prevodnik, Bianca C Reisdorf

**Affiliations:** University of Ljubljana, Faculty of Social Sciences, Ljubljana, Ljubljana, Slovenia; University of Ljubljana, Faculty of Social Sciences, Ljubljana, Ljubljana, Slovenia; Masaryk University | Faculty of Social Studies, Brno, Jihomoravsky kraj, Czech Republic; University of Ljubljana, Ljubljana, Ljubljana, Slovenia; University of North Carolina at Charlotte, Charlotte, North Carolina, United States

## Abstract

The COVID-19 pandemic has had profound implications on how older adults engage with the digital world. While there is converging evidence that during the pandemic the number of online newcomers has increased the most among older adults, digital inequalities have become even more condensed among the socially disadvantaged groups in this period. Importantly, such patters of age-related digital exclusion were not observed only in terms of self-reliant internet use but also in older adults’ ability to access internet resources indirectly by asking others to act on their behalf – a practice known as use-by-proxy. Drawing on the van Dijk’s resources and appropriation theory, we aim to understand which categorical and resource inequalities determine the direct and indirect forms of internet use among older adults during the pandemic. Therefore, in November 2021 a telephone survey (N = 701) was carried out among individuals aged 65+ in Slovenia. Three logistic regression models were fitted to explore the association between socio-demographic characteristics of respondents and their access to material and social resources with internet use as well as with availability and activation of use-by-proxy. The results suggested that both categorical and resource inequalities are predictors of internet use. Conversely, availability of use-by-proxy is strongly associated only with social resources, while use-by-proxy activation depends on material resources and household composition. Overall, we argue that social inequalities affect also the indirect forms of internet use in later life which means that the ability to compensate by help for digital inequalities is also stratified unevenly.

